# Air pollution seasons in urban moderate climate areas through big data analytics

**DOI:** 10.1038/s41598-024-52733-w

**Published:** 2024-02-06

**Authors:** Mateusz Zareba, Elzbieta Weglinska, Tomasz Danek

**Affiliations:** Department of Geoinformatics and Applied Computer Science, Faculty of Geology, Geophysics and Environmental Protection, AGH University of Krakow, Adama Mickiewicza 30, 30-059 Krakow, Malopolska Poland

**Keywords:** Climate sciences, Environmental sciences, Engineering, Mathematics and computing

## Abstract

High particulate matter (PM) concentrations have a negative impact on the overall quality of life and health. The annual trends of PM can vary greatly depending on factors such as a country’s energy mix, development level, and climatic zone. In this study, we aimed to understand the annual cycle of PM concentrations in a moderate climate zone using a dense grid of low-cost sensors located in central Europe (Krakow). Over one million unique records of PM, temperature, humidity, pressure and wind speed observations were analyzed to gain a detailed, high-resolution understanding of yearly fluctuations. The comprehensive big-data workflow was presented with the statistical analysis of the meteorological factors. A big data-driven approach revealed the existence of two main PM seasons (warm and cold) in Europe’s moderate climate zone, which do not correspond directly with the traditional four main seasons (Autumn, Winter, Spring, and Summer) with two side periods (early spring and early winter). Our findings also highlighted the importance of high-resolution time and space data for sustainable spatial planning. The observations allowed for distinguishing whether the source of air pollution is related to coal burning for heating in cold period or to agricultural lands burning during the warm period.

## Introduction

Air pollution is an important factor affecting general public health. It has been proved that overexposure to PMx may lead to neurodegeneration diseases like Alzheimer’s and Parkinson’s^1^. In fact, air pollution may secondarily lead to many problems including even the failure of health care systems as patients with neurodegeneration problems often need full-time, professional care, particularly within Europe, known as the globe’s most extensive aging population^2^. Exposure to polluted air can cause also respiratory problems including bronchitis and asthma^3^. What more can also increase the risk of heart disease and even stroke. Recent studies show that even relatively poorly urbanized areas with episodically exceeded air quality standards may contribute to an increase in the number of ischemic strokes^4^. Depending on exposure time to polluted air it may be also a key factor leading to lung cancer^5^, and chronic respiratory problems^6^. Air pollution can aggravate existing health conditions, such as diabetes and mental health disorders. Air pollution is not only a problem for public health, but it also affects the environment and wildlife. The polluted air can harm crops, forests, and water, and in consequence affect wildlife by altering their natural habitat. Air pollution can make it more difficult for plants, animals^7^, and even humans^8^ to reproduce, resulting in long-term damage to entire ecosystems. This can lead to a decline in biodiversity, and a decrease in the overall health of the planet. Air pollution also plays a role in accelerating climate change, which can have even more severe consequences for the environment and wildlife^9^. Air quality and climate are inseparably linked^10^. Many sources that pollute the air are the source of greenhouse gases that affect the climate^11^. These pollutants, through their effects on solar and terrestrial radiation, lead to climate change^12^.

Krakow is located in southern Poland, within the moderate climate zone of central Europe which means that typical four astronomical seasons are observed—spring, summer, autumn, and winter. Many climatologists have attempted to separate the thermal seasons in Poland, due to the fact that the division by calendar months does not reflect the seasonality of the climate^13^. The 6 thermal seasons in Poland are distinguished by Guminski’s method^14^ based on average daily air temperature. Thermal summer occurs when the average daily air temperature exceeds 15 ^∘^C. Thermal spring and autumn occur when the average daily temperature ranges from 5 to 15 ^∘^C. Negative daily average temperatures occur in winter. This method allows for distinguishing two additional seasons: early spring (przedwiosnie) and pre-winter (przedzimie), and they are characterized by a temperature of 0^∘^–5^∘^. Based on our observation, if we look at particulate concentrations, we can distinguish only two seasons: warm and cold.

The unique topography of the Krakow area significantly hinders both vertical and horizontal natural air circulation^15^. The study^16^ demonstrates that the influence of foehn winds on Kraków’s air pollution is highly dependent on the interaction between the winds and the city’s geographical features, leading to distinct variations in PM10 concentrations and air quality throughout various regions of the city. The study by Danek et al.^17^ expanded upon this research by implementing a complex geostatistical approach to analyze PM concentrations in both space and time, highlighting a correlation between topographical features, meteorological conditions, and particulate matter levels.Additionally, the study highlights Krakow’s disadvantageous geographical position, which contributes to its tendency to accumulate pollutants from surrounding areas. Previous works by Danek et al.^18^ also conducted a historical analysis of PM2.5 concentrations to show the effect of changes in clean air regulations in Krakow. The other research demonstrates that the combination of a solid fuel combustion ban and COVID-19 lockdown measures significantly altered the characteristics of air pollution in Krakow, leading to a marked decrease in PM2.5 concentrations and changes in the composition of air pollutants^19^. Understanding the dynamics of particulate matter concentration is essential for evaluating its impact on public health and the environment. This has been demonstrated through numerous studies in urban settings, including Krakow. In the previous study^20^ unsupervised machine techniques were utilized to examine the spatiotemporal distribution of air pollution, employing PM10 data gathered hourly from sensors across Krakow over a year. By applying clustering methods, the study uncovered significant disparities in the average and peak concentrations of pollutants.

Big data refers to extremely large and complex sets of data constantly growing through data collection from various sensors in real-time. These sets are often multi-dimensional and multi-domain, which creates difficulties in their processing and statistical analysis. They are incredibly useful in observing phenomena that were not previously observable due to a lack of sufficient observations to accurately determine certain patterns of a given phenomenon^21^. A dense grid of LCS was used in this study. These sensors were equipped to measure various environmental parameters, including PM1, PM2.5, PM10, temperature, humidity, and pressure. Data were acquired at a temporal resolution of 1 h during the whole year, yielding a total of 985,000 unique records. The resultant large dataset enabled the examination of annual variations in these environmental parameters at a high spatial resolution, thereby providing valuable insights into the air quality of the urban area under investigation. The analysis of big data allows for conclusions that would be difficult to achieve with standard data analysis techniques.

There were studies connecting climate and atmosphere physical components focusing on future mortality related to air pollution and climate changes^22^ or focusing on the connection between meteorological factors and aerosol optical depth^23^. The novelty of the presented study is to show the local climate impact on air pollution using long-term observations in very high time and space resolution. This research will demonstrate the application of big data analysis to determine annual patterns between climate and air pollution in urban areas using a dense, high-resolution grid of measurements. By utilizing additional meteorological data, it will be possible to identify whether the impact of specific meteorological components on levels of particulate matter pollution varies on an annual basis. This innovative approach to data analysis in the context of air pollution will enable better urban planning. It was hypothesized that: (1) big data can be used to analyze patterns between climate components and air pollution, (2) the impact of meteorological factors on air pollution levels varies during different seasons of the year, (3) the seasons identified based on climate analysis may not reflect fluctuations in pollution levels in an urban context, and (4) the influence of neighboring towns on pollution in Krakow is particularly significant during colder months.

## Methods

### Data source and validation

**Figure 1:**
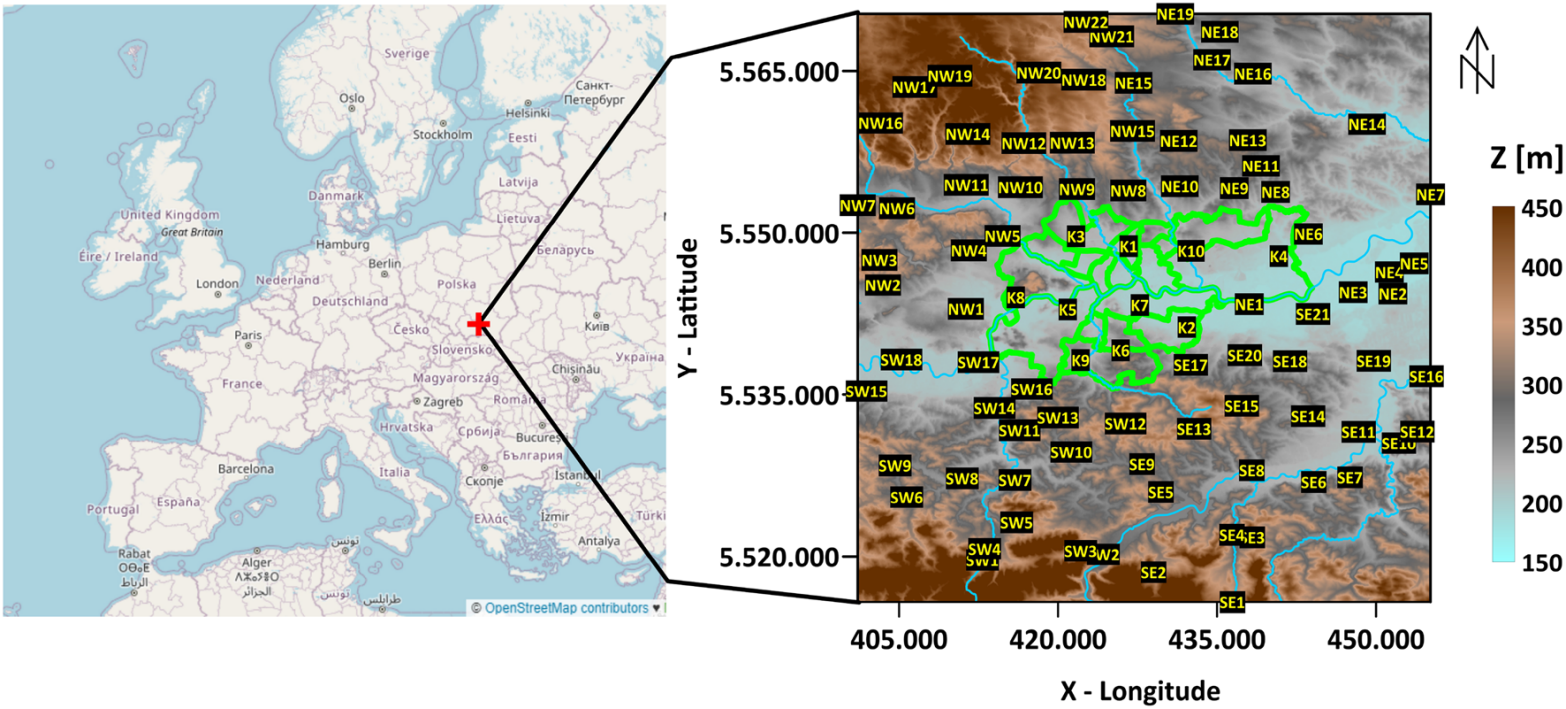
Digital terrain model of Krakow (grey lines—city districts borders) and its surrounding with airly sensor locations (ID in black) together with the rivers (blue lines). Data sources: Digital terrain—European Union, Copernicus Land Monitoring Service 2022, European Environment Agency (EEA); Europe Map—OpenStreetMap^24^.

Krakow is situated in the valley of the Vistula River, which bisects the city on a latitudinal axis. The Sandomierz Basin is located to the west of the city, while the Oswiecim Basin is situated to the east. The air mass movement is affected by Polish Jurassic Highland on the north side and the Wielickie foothills on the south^25^. Danek et al.^17^ proved that the terrain influence is a key factor for air pollution migration to the city from neighboring cities. Polish legislation follows general European Union law described in the Ambient Air Quality and cleaner air for Europe Directive no. 2008/50/EC^26^. The allowed concentration for PM2.5 is 25 $\upmu$g/m^3^ (1-year average) and 50 $\upmu$g/m^3^ (24-h average) for PM10. Reference measurements are carried out according to the PN-EN12341 and PN-EN 16450 norms. Measurements are publicly available through Chief Inspectorate for Environmental Protection website (Chief Inspectorate for Environmental Protection, 2021). Unfortunately, there are less than 300 reference measurement stations in Poland. This is not enough to provide reliable, high-resolution spatial observations. In this study, the 1-year observations of 90 low-cost sensors (LCS) provided by Airly (https://map.airly.org) were used. All sensors were localized in Krakow city and its closest urban neighborhood. Their high accuracy related to the reference measurements was proved by Danek and Zareba^18^. It has been shown that the main source of air pollution during the autumn-spring period is household coal combustion in the neighborhood as Krakow itself entered the pro-clean law forbidding the use of coal for heating^27^. The second main source of PMs’ carbon fraction is transportation, which is most significant during the summer months, and less during the rest of the year. The third main source is natural combustion (biogenic fraction), which concentration is constant throughout the year^28^. In this experiment, 90 LCS optical Airly sensors located in Krakow and its neighborhood were utilized (Fig. 1). These sensors enable the measurement of three main PMx fraction concentrations, as well as temperature, humidity, and pressure. Some of them are also capable of measuring NOx, CO2, and Ox^29^. In this study, we analyzed PM1, PM2.5, and PM10, in conjunction with meteorological factors that were available on each sensor. According to Airly’s statement, the accuracies of measurement are PM1: 5 $\upmu$g/m^3^ (in the range 0–100 $\upmu$g/m^3^) and 10 $\upmu$g/m^3^ (over 100 $\upmu$g/m^3^); PM2.5: 10 $\upmu$g/m^3^ (in the range 0–100 $\upmu$g/m^3^), 10% (in the range 101–500 $\upmu$g/m^3^), 20% (over 500 $\upmu$g/m^3^); PM10: same accuracy as PM2.5. The compatibility of measurements made by these sensors with reference measurements during the studied period has been shown in Danek and Zareba’s^18^ study. Data was acquired from the beginning of spring 2021. In this study, a 1-year observation period between March 2021 and March 2022 was investigated, with almost 1 million unique observations. The sensors in the studied area were strategically placed to create a regular measurement grid using a custom-made algorithm in R. Information about average wind speed was collected from E-OBS gridded dataset^30^. As per the WMO document^31^, it is evident that uncertainties within LCS measurements surpass those found in reference stations. For instance, the standard precision for gravimetric measurements stands at 2 $\upmu$g/m^3^^26^, while the manufacturer of Airly sensors indicates a precision level of 10 $\upmu$g/m^3^ for PM10, marking a significant fivefold difference. Although LCSs being sensitive to atmospheric factors, the data provided by Airly is adjusted, yet the complete uncertainty of individual sensors remains undisclosed. To assess the precision of LCS sensors, adhering to Level-4 LCS data processing standards, outlined in the WMO document by Peltier et al.^31^, a comparative analysis was conducted. The measurements from two distinct LCS sensors close to their respective official government reference stations were used. The selection criteria for these stations aimed to encapsulate the broad geographical and urban spectrum within the analyzed region. For Krakow city’s urban locale, the Krakow–Bujaka reference sensor conducted continuous automated measurements and was compared with the nearby LCS K5 sensor. Similarly, in the densely forested terrain of Puszcza Niepolomicka, a reference sensor located near 3 Maja Street in Niepolomice, also conducting continuous automated PM10 measurements, was compared with the LCS SE21 sensor. To ensure comparability between LCS-type and reference sensors, measurements were averaged over a 24-h period, represented by a 24-sample window. These averaged measurements, covering the period from September 2021 to September 2022, facilitated the generation of cross-plots and the calculation of Pearson correlation coefficients. Subsequently, the differences between the measurements of the reference sensor and the neighboring LCS sensor were delineated, enabling daily tracking of concentration discrepancies. Additionally, to reveal weekly trends in differences between reference stations and the nearest LCS, a Seasonal-Trend decomposition using LOESS (STL) method^32^ was employed, generating trend curves within 7-days intervals.

**Figure 2:**
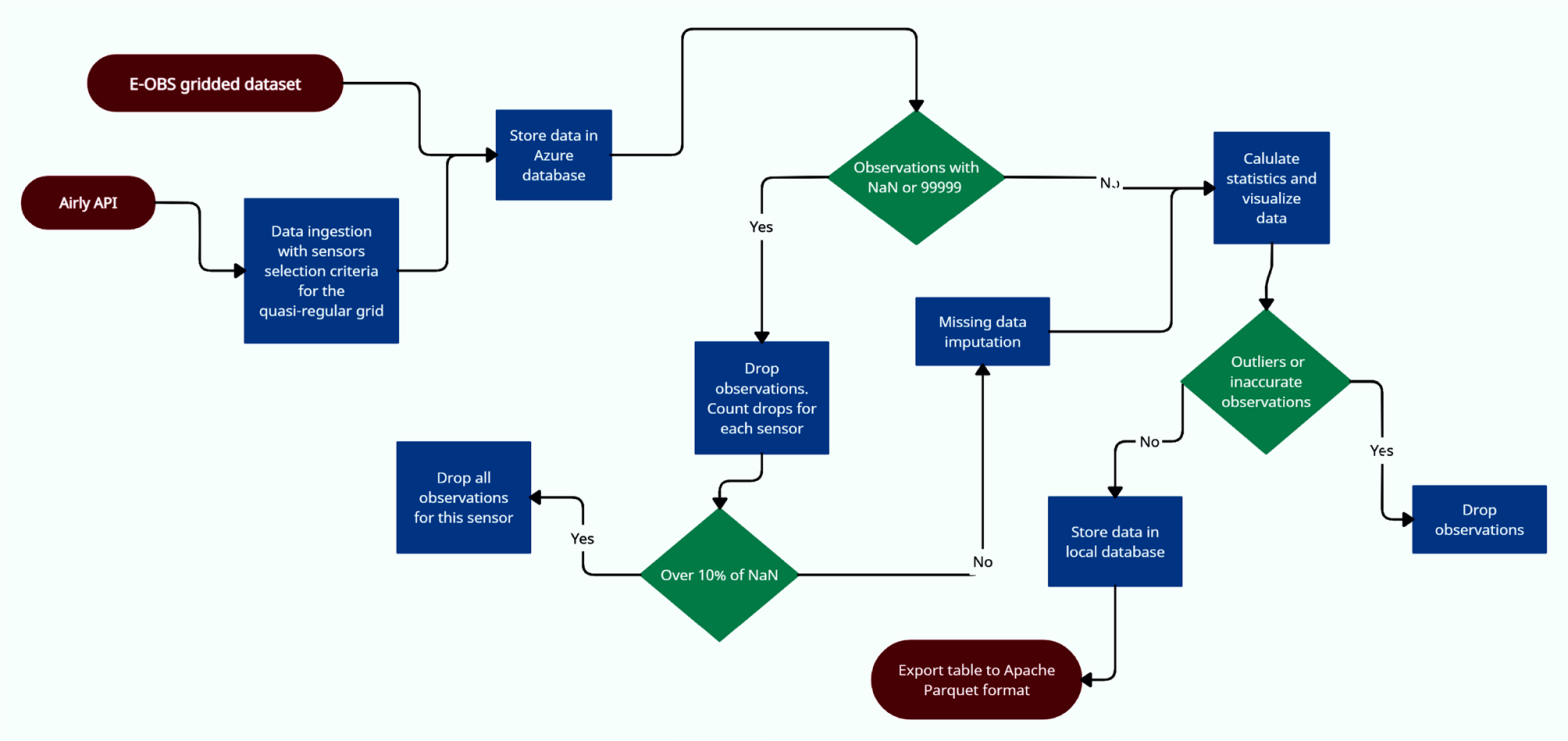
Big data workflow.

Proper preparation of a big data pipeline is an important and multi-step process. This study involved data collection from two different sources—Airly API and E-OBS dataset using the R programming language. Data ingestion and the pre-processing pipeline is shown in Fig. 2. After the initial check (for not numeric or over-scale observations) dataset was stored in the Microsoft Azure cloud database. The further processing, analysis, and visualization included only data for sensors with over 90% valid observations in the investigated time period. The method of addressing missing data for indicators in which missing values did not exceed 10% involved the use of K-Nearest Neighbour Imputation^33^. The second round of quality checks consisted of statistical tests and distribution visualization to ensure data quality by removing outliers and observations with data drifts. The final dataset was exported to open-source Apache Parquet format which is optimized for storing big, complex, tabular datasets at scale through the implementation of efficient data compression and encoding techniques. The dataset from the period of March 2021–March 2022 was subsequently processed using the Python programming language and the ArcGIS software. A mask was applied to the studied area, allowing for separate visualization of data within the city of Krakow and its northwest, northeast, southwest, and southeast neighborhoods. This will allow for observation of changes in the town itself and its surroundings. This division was based on Danek et al.^17^ research. Indicators of PM1, PM2.5, and PM10 concentrations show general two annual trends, therefore, a division into a warm and cold period was made. No tendency to form separate trends for astronomical or calendar seasons in the region was observed. Research in this area shows that the critical element contributing to the formation of the particular matter is the relative temperature perception^27^. The division into a warm and cold period can be related to the thermal division of seasons by Guminski’s method. The next stage was to present the averages and maximum concentrations of particulate matter, specifically PM10, according to the aforementioned division into the city of Krakow and surrounding areas for the entire year, warm and cold periods. This was achieved using visualizations on graphs. Maps were created showing the distribution of maximum and average PM10 concentrations in each month of the studied period. The relationships between meteorological indicators and PM were analyzed on an annual basis, according to astronomical seasons and divisions into warm and cold periods. A kernel density estimate (KDE) plot was also performed for temperature. The average wind speed was analyzed in the studied period.

## Results and discussion

### LCS sensors measurements evaluation

**Figure 3:**
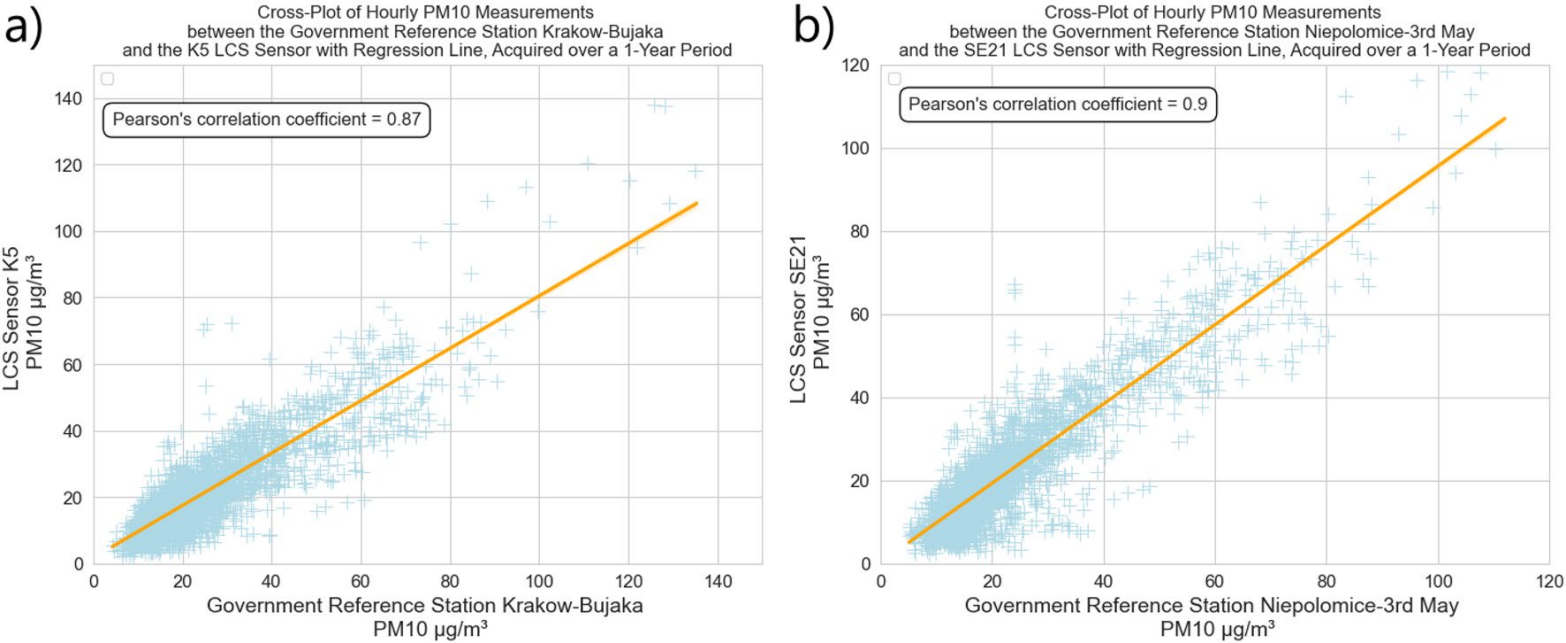
Cross-plot of PM10 signals from the Government Reference Station (GRS) and near LCS sensor measurements (light blue) with regression line (orange): (**a**) GRS—urban area Krakow–Bujaka and LCS K5; (**b**) GRS—forest area Niepolomice-3rd May and LCS SE21.

**Figure 4:**
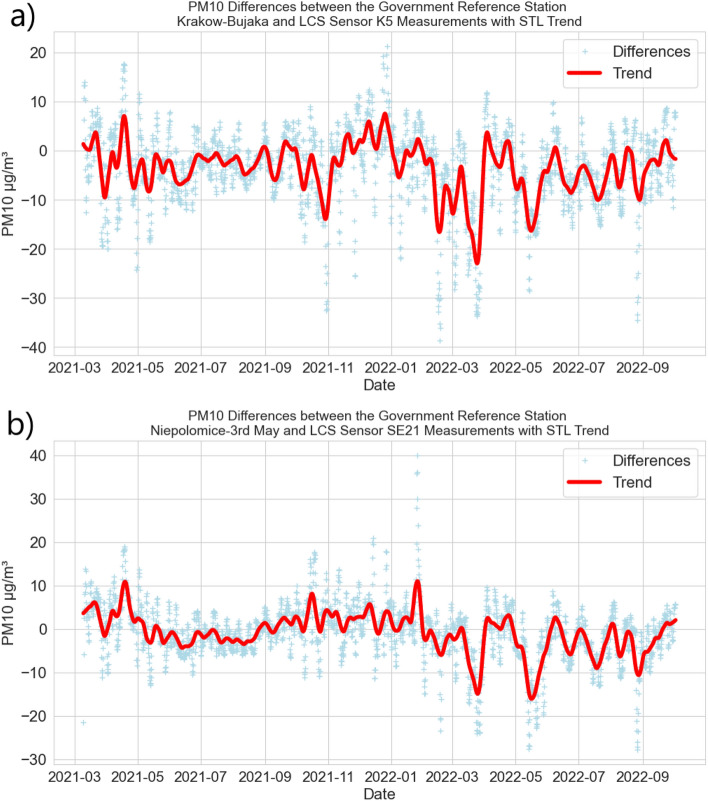
PM10 differences between the Government Reference Station (GRS) and near LCS sensor measurements (light blue) with 7-day STL trend (red): (**a**) GRS—urban area Krakow–Bujaka and LCS K5; (**b**) GRS—forest area Niepolomice-3rd May and LCS SE21.

Figure 3a presents the correlation results between measurements from the governmental reference station in Krakow city and the LCS sensor K5 positioned near that station. Figure 3b depicts a similar comparison for a sensor located in the area of 3rd Maja Street in Niepolomice alongside sensor SE 21. For both sensors, the Pearson correlation coefficient for annual observations is notably high, standing at 0.87 for the urban area and 0.9 for the forest-dominated area. Analysis of the differences, as shown in Fig. 4a,b, indicates that LCS sensor measurements generally align closely with the readings from reference stations, falling within the accuracy range declared by the Airly sensor manufacturer. However, there are more notable discrepancies for the sensor placed in the urban area of Krakow, which is an expected phenomenon. Urban structures and traffic dynamics can significantly influence local changes in pollution levels. It is important to note that the closest station for comparison is not precisely positioned in the same location as the reference station. Interestingly, both the sensors in the forested and urban areas exhibit similar weekly trend characteristics. The smallest differences occur in the months from May to September 2021, followed by only slightly larger differences until January 2022. Two weeks stand out from this trend—one at the end of March 2022 and one in the middle of May 2022. These differences consistently average at a maximum of 12–15 $\upmu$g/m^3^ each week. According to the WMO report and Level-4 LCS data processing standards^31^, these results are considered adequate for spatial analyses, given their high similarity to the closest reference stations’ measurements. Airly also utilizes its accuracy analysis tools, incorporating readings from reference stations and other sensors through machine learning techniques.

### PM and meteorological factors

**Figure 5:**
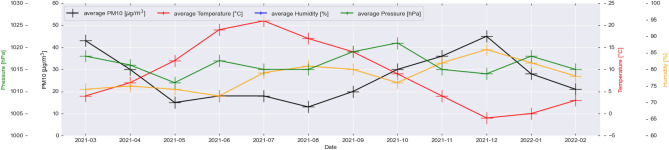
Monthly averages of PM10 (black) and meteorological factors: pressure (green); temperature (red); humidity (yellow). Values calculated based on all available observations.

**Figure 6:**
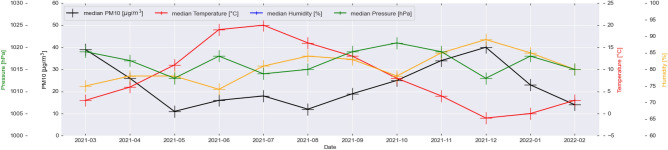
Monthly median of PM10 (black) and meteorological factors: pressure (green); temperature (red); humidity (yellow). Values calculated based on the all available observations.

**Figure 7:**
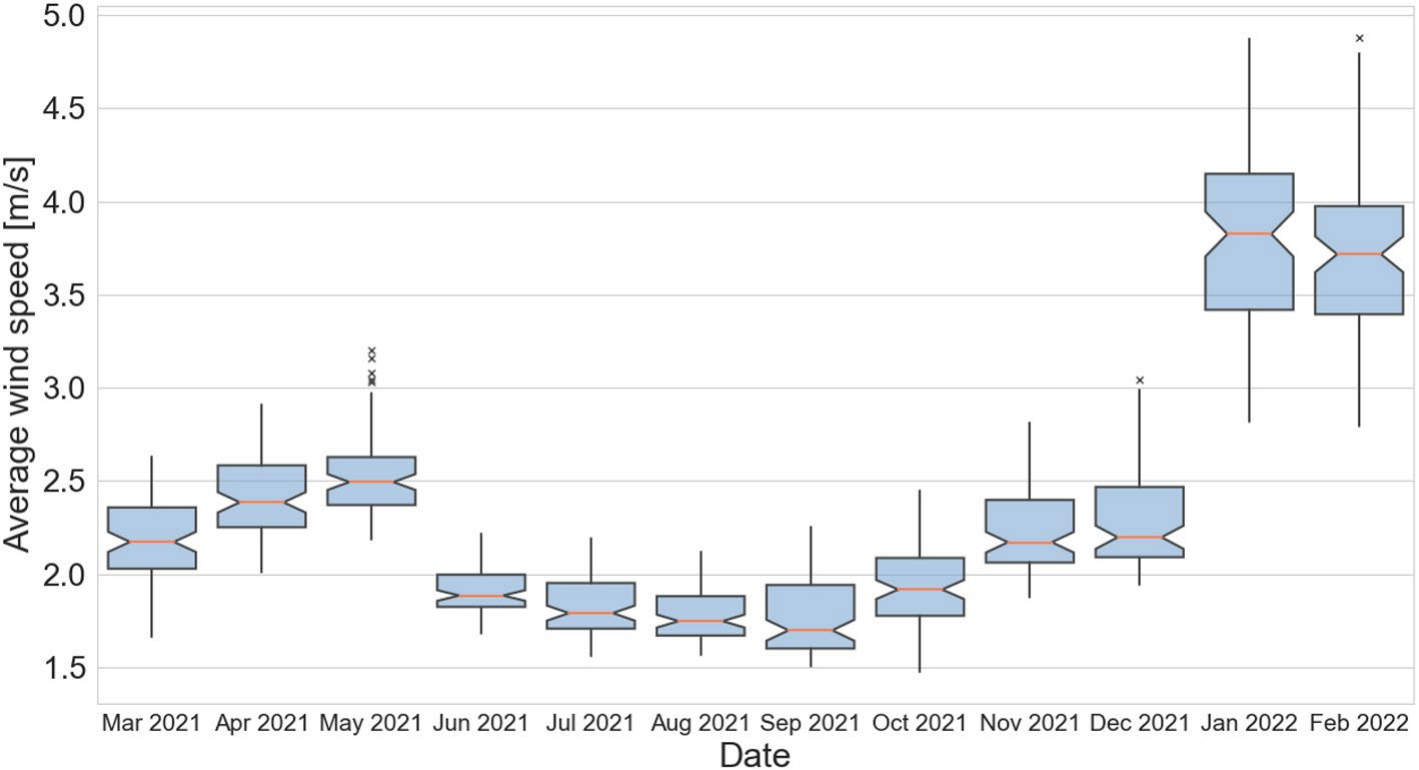
Box plots of average wind speed in investigated region.

Figure 5 shows the monthly average values of pressure, temperature, humidity, and PM10, while Fig. 6 shows the same set of parameters for the monthly median readings in Krakow and its surroundings. Clearly, three 4-monthly humidity cycles with a hyperbolic characteristic can be seen. In the case of pressure, four cycles of different lengths can be distinguished. Temperature and PM10 are characterized by two main cycles with opposite characteristics. With an increase in temperature, a decrease in average and most frequent values of PM10 is observed. The points of intersection of the temperature and PM10 curve occur in the middle of April and October. According to Guminski’s thermal seasons’ division, it is possible to divide the year into eight thermal seasons. It can be observed that the reversal of trends between temperature and PM10 occurs in the months when the average temperature is around 5 ^∘^C. This includes the following seasons: pre-winter, winter, and pre-spring. For further analysis, the year is divided into two warm periods—covering the period from the beginning of October to April, and the warm period from the end of April to October. Interestingly, both monthly median and average concentrations values do not show that the daily concentration standards have been exceeded, yet on many days in the cool period, Krakow is among the world’s most polluted cities. Figure 7 presents the average values of wind speed in all sensors’ locations in the form of a box plot (also known as a box-and-whisker plot). Data for average wind speeds have been presented in a separate figure, due to the fact that these are data collected for monthly averages in each receiver separately. Other meteorological parameter values were collected in LCS sensors in hourly windows. An interesting phenomenon is that the lower the speed, the more compact and less symmetrical the wind speed distribution is in the city and neighborhood. This means that there are places where the wind blows at much higher speeds than in others. This may cause locally that the air in these places is better. Worryingly, despite relatively high wind speeds in the cold period, this does not lead to much better air quality in this period. On the one hand, the wind in this area has a positive impact by ventilating the city, but at the same time, it is one of the factors pushing pollution through the western terrain depression (the dominant wind direction is west). In some months, it is seen that as the average speed increases, the average pollution decreases (March–May 2021), while from August to December 2021, the opposite trend is seen—as wind speed increases, average concentrations increase. An important observation of all the mentioned meteorological factors is that in the case of cities located in the moderate climate zone, where the main component of the PM10 carbon fraction is coal burning, the largest and most direct relationship between average concentrations is between temperature, not average wind speed in the area. Our observations about wind align with Bokwa’s^15^ study, which found that in Krakow, winds are generally weak with the majority of wind directions being west to east. These observations are in line with other studies related to the importance of meteorological factors and physical components of atmosphere^34^ including even the COVID-19 analysis^35^.

**Figure 8:**
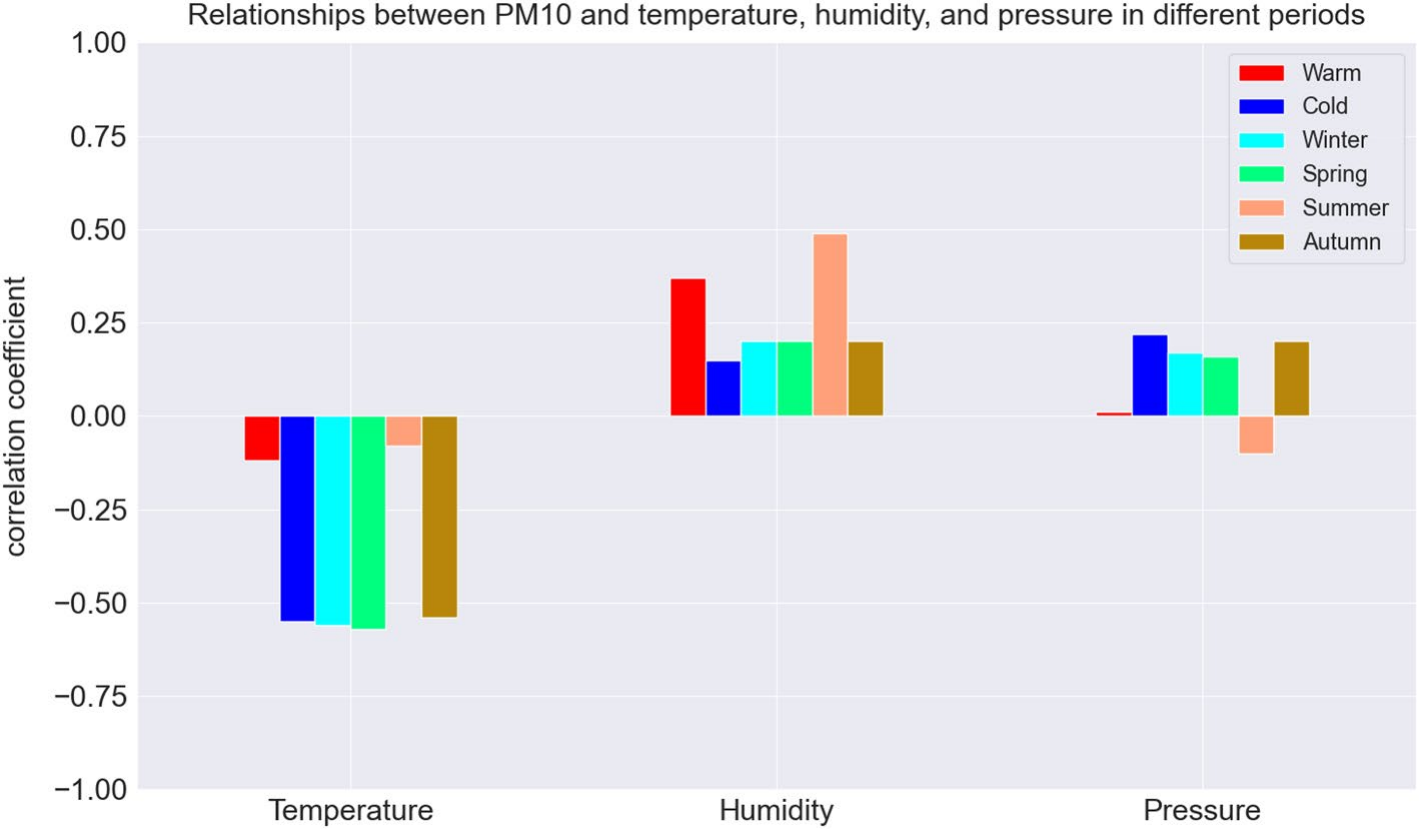
Relation between meteorological factors (temperature, humidity, and pressure) and PM10 calculated for all observations in the following periods: astronomical summer (salmon), autumn (brown), winter (light blue), spring (green); and in pollution seasons: warm (red), cold (dark blue).

**Figure 9:**
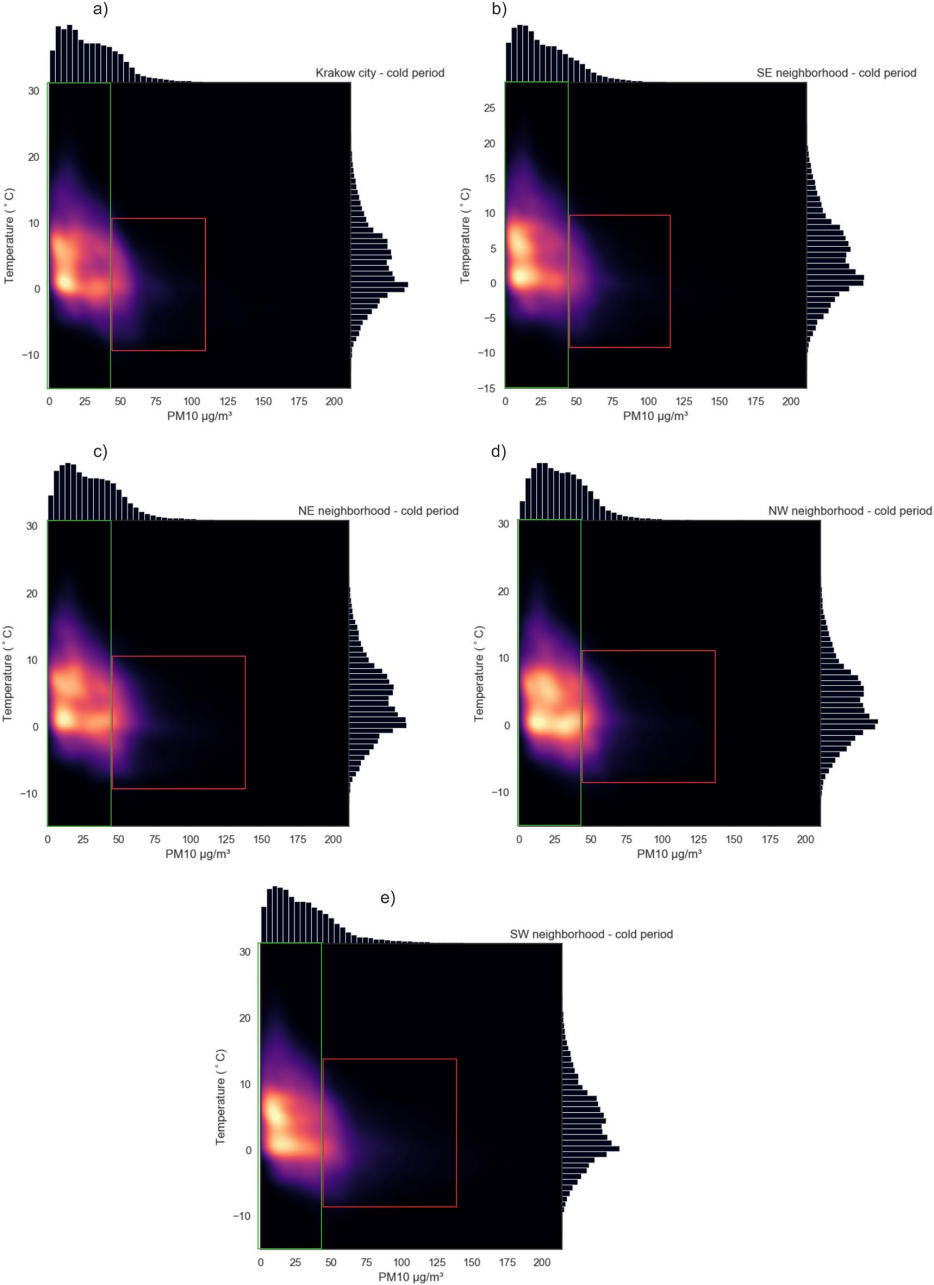
Kernel density estimate plots of PM10 and temperature in cold season for (**a**) Krakow city; (**b**) southeastern region; (**c**) northeastern region; (**d**) northwestern region; (**e**) southwestern region. Green rectangle represents the allowed air pollution level, red one represents exceeded concentrations.

Analyzing the values of Pearson’s correlation coefficients between PM10 and individual meteorological factors measured by Airly sensors (Fig. 8), it can be stated that during the warm and summer periods, the indications show the highest positive correlation with humidity (Pearson’s correlation coefficient = 0.5), the least dependence on humidity can be seen in the winter period (Pearson’s correlation coefficient = 0.2). In the case of pressure, no dependence can be seen in the warm period, in the remaining periods the Pearson’s correlation coefficient never exceeds an absolute value of 0.2. In winter, fall, and spring, a positive relationship is visible, while in summer it is inverse. This is logical in accordance with the principles of atmospheric circulation. When the pressure is high, cloudiness is usually not observed. In winter, cold months will favor low-temperature episodes, due to the rapid loss of heat, while in summer, when the day is long and solar radiation is intense, we will observe warm days. Without a doubt, the most significant relationship exists between temperature and PM10 indications. This is particularly visible in the winter period and in the case of astronomical winter, spring, and fall. Interestingly, the greatest relationship (Pearson’s correlation coefficient = 0.58) is observed in spring. Observations from Krakow show a similar reverse relationship between air pollution from fossil fuel heating and the temperature presented by Ambade et al.^36^, but there is no similarity between other meteorological parameters showing positive relation. The reason can be related to the different climate specifications. In Fig. 9, KDEs for temperature and PM10 in Krakow and individual regions around the city are presented, separated by warm and winter periods. The green rectangle marks the standard air quality indications, and the red concentrations exceed it. It is clearly visible that pollution outside the norm is generated in the temperature range from − 10 to 10 ^∘^C with a clear maximum for temperatures around 0. This is related to relative thermal sensations of cold and consistent with previous observations^18,37^.

### PM1, PM2.5, and PM10 annual concentrations

**Figure 10:**
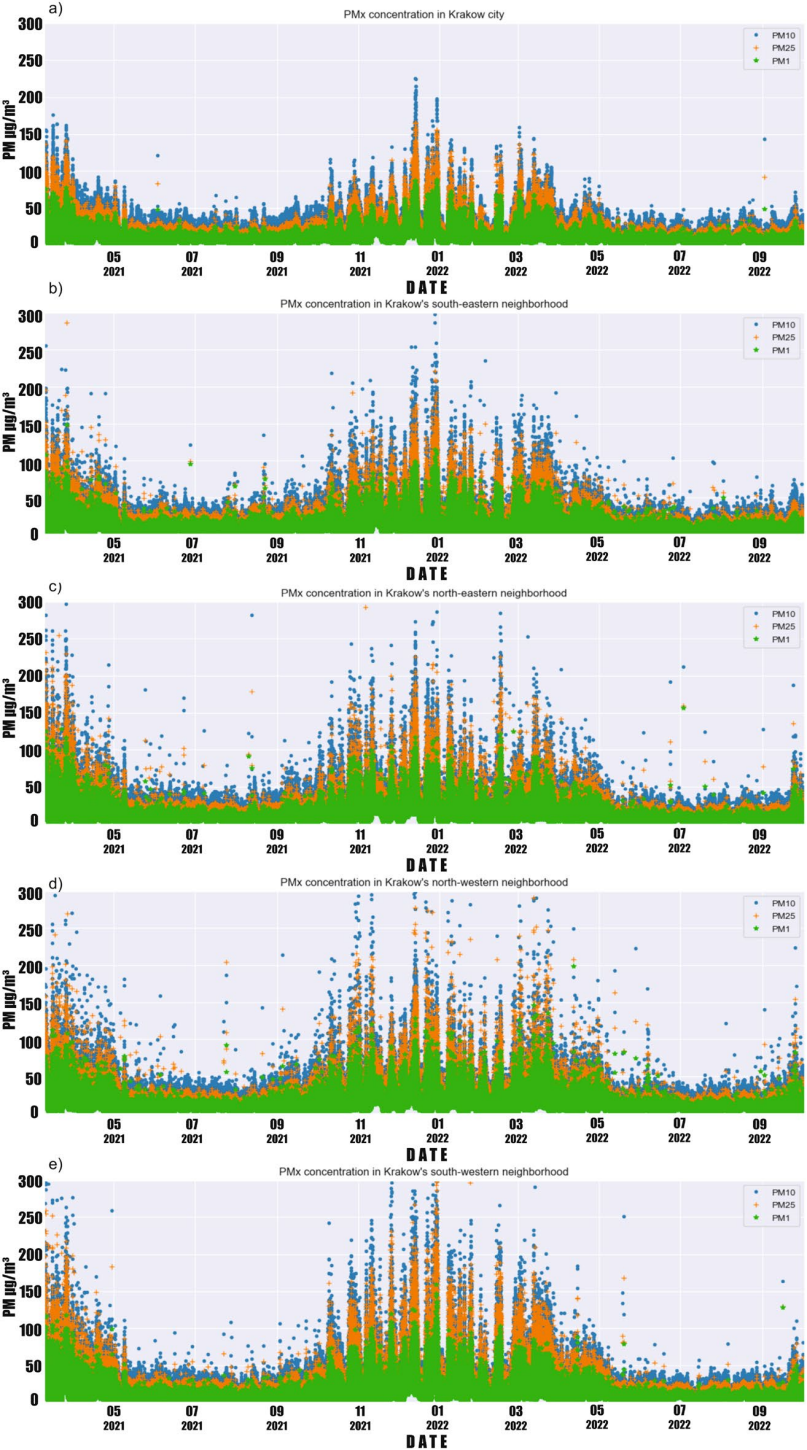
PM1, PM2.5, and PM10 concentrations (March 2021–February 2022) in: (**a**) Krakow city; (**b**) southeastern region; (**c**) northeastern region; (**d**) northwestern region; (**e**) southwestern region.

**Figure 11:**
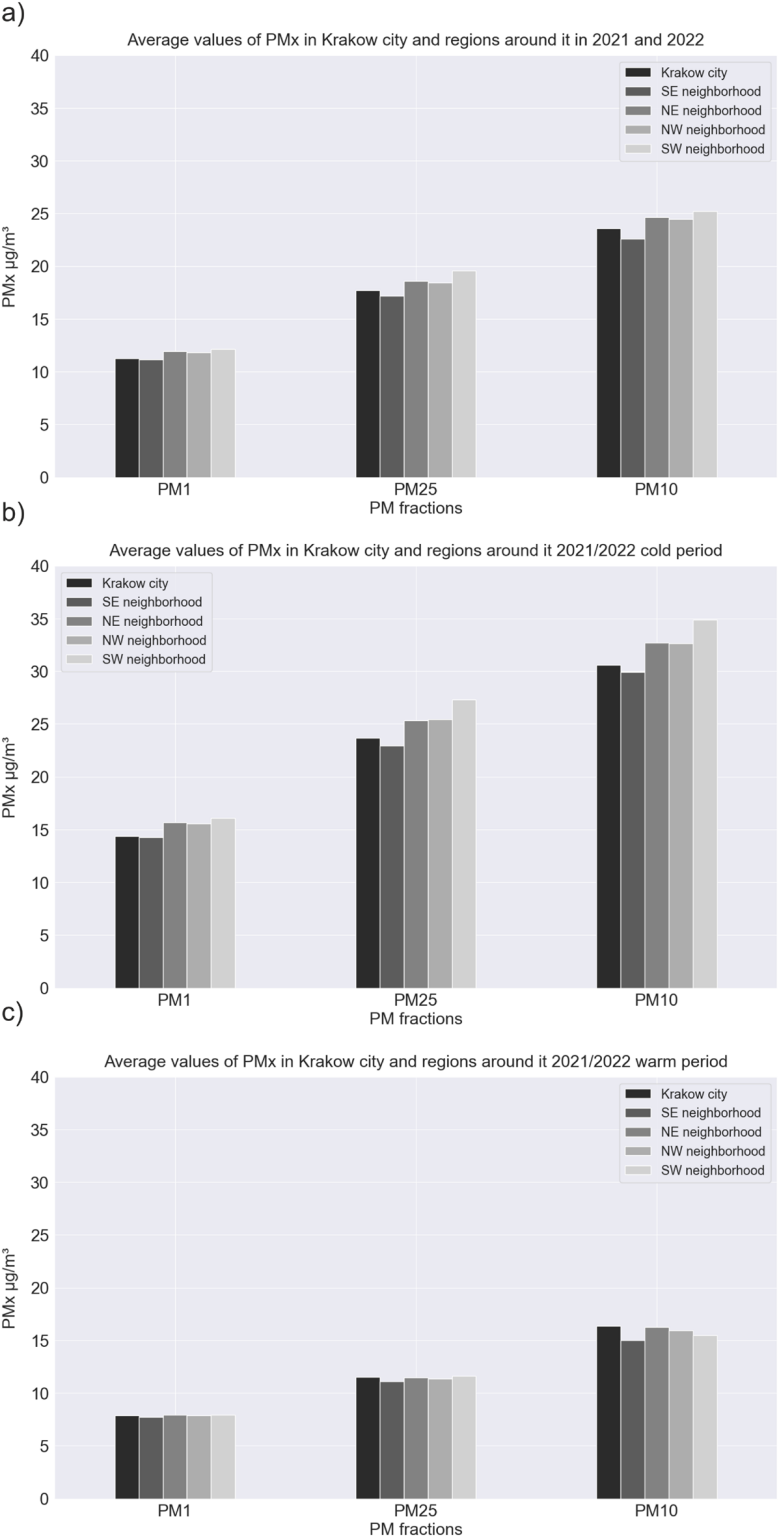
The average values of PM1, PM2.5, and PM10 in Krakow city and regions around the city for (**a**) 1-year period; (**b**) cold period; (**c**) warm period.

**Figure 12:**
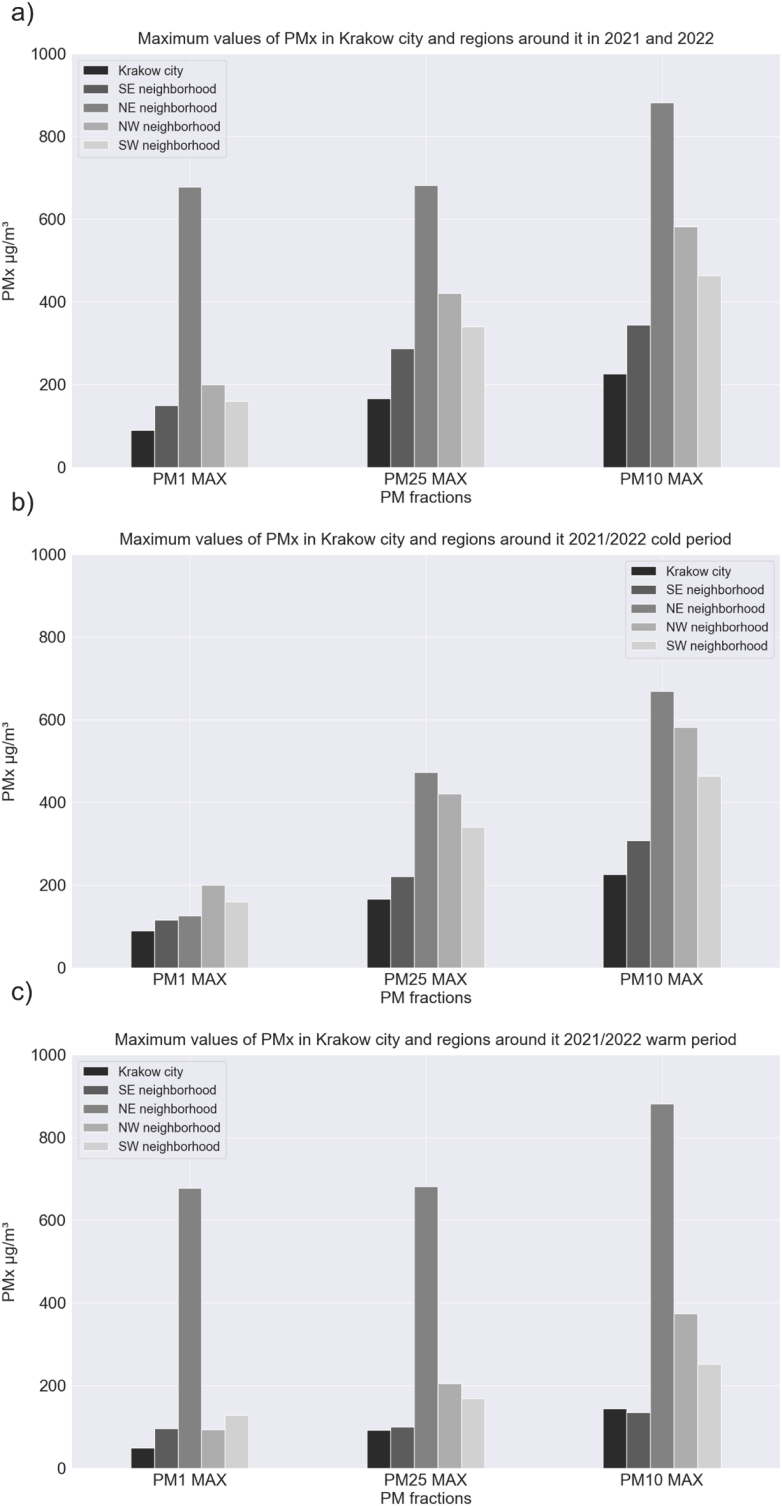
The maximum values of PM1, PM2.5, and PM10 in Krakow city and regions around the city for (**a**) 1-year period; (**b**) cold period; (**c**) warm period.

Figure 10 presents hourly values of all observations analyzed in the studied period, divided into 4 regions around Krakow and in the city itself. A clear stratification between measurements of various particulate matter (PM10, PM2.5, and PM1) is visible. This is expected, as each larger fraction also contains particles of smaller fractions. It is clearly visible that the surrounding regions have significantly more high-emission episodes. In Krakow, there are practically no readings above 250 $\upmu$g/m^3^, which cannot be said about the surrounding regions, where readings around 300 $\upmu$g/m^3^ occurred relatively frequently. The most high-emission episodes can be observed in the northern regions. Interestingly, in the city of Krakow during the warm period, there are practically no values deviating from the trends, which cannot be said about the surrounding towns. In the northeastern and northwestern regions, there are readings even reaching 200 $\upmu$g/m^3^ in the summer months, and close to 300 $\upmu$g/m^3^ in September. This local emission may indicate the burning of materials or fuels other than coal (used for heating in cold periods). The media has frequently reported on the practice of burning grass and agricultural land in both the country and the district around Krakow city^38^. It is worth mentioning that this practice is strictly prohibited by law and subject to financial penalties. Figure 11 presents bar plots of the average values of various particulate matter fractions, divided by regions and periods—annual, warm, and cold. Figure 12 presents a similar set of charts, but for maximum readings. The average values show an approximately linear trend of increasing values depending on the fraction in each group in the annual and winter period. In the summer period, this dynamic is smaller. Looking at the overall ratio of PM10 to PM2.5, it can be said that in the cold period, we are dealing with anthropogenic dust from coal burning^17,39–42^. The highest average concentrations are measured in the southwestern region and the lowest in the southeastern region for each fraction. The cause of the lowest average observations may be related to the occurrence of a vast green area of Puszcza Niepolomicka in this part of the investigated area. In the warm period, low values of PMx were maintained in each group, with the lowest readings again occurring in the southeastern group. In the case of maximum PMx observations, the situation looks slightly different. The highest values always occurred in the northeastern region. Interestingly, it is where strong emission episodes (significantly above 300 $\upmu$g/m^3^) occurred in the warm period. This may be related to the aforementioned grass and agricultural land burning. In this region, there are practically no forests, and a larger part of the area is occupied by meadows, agricultural land, or single-family housing. The lowest maximum concentrations for each fraction occurred in the city of Krakow itself.

**Figure 13:**
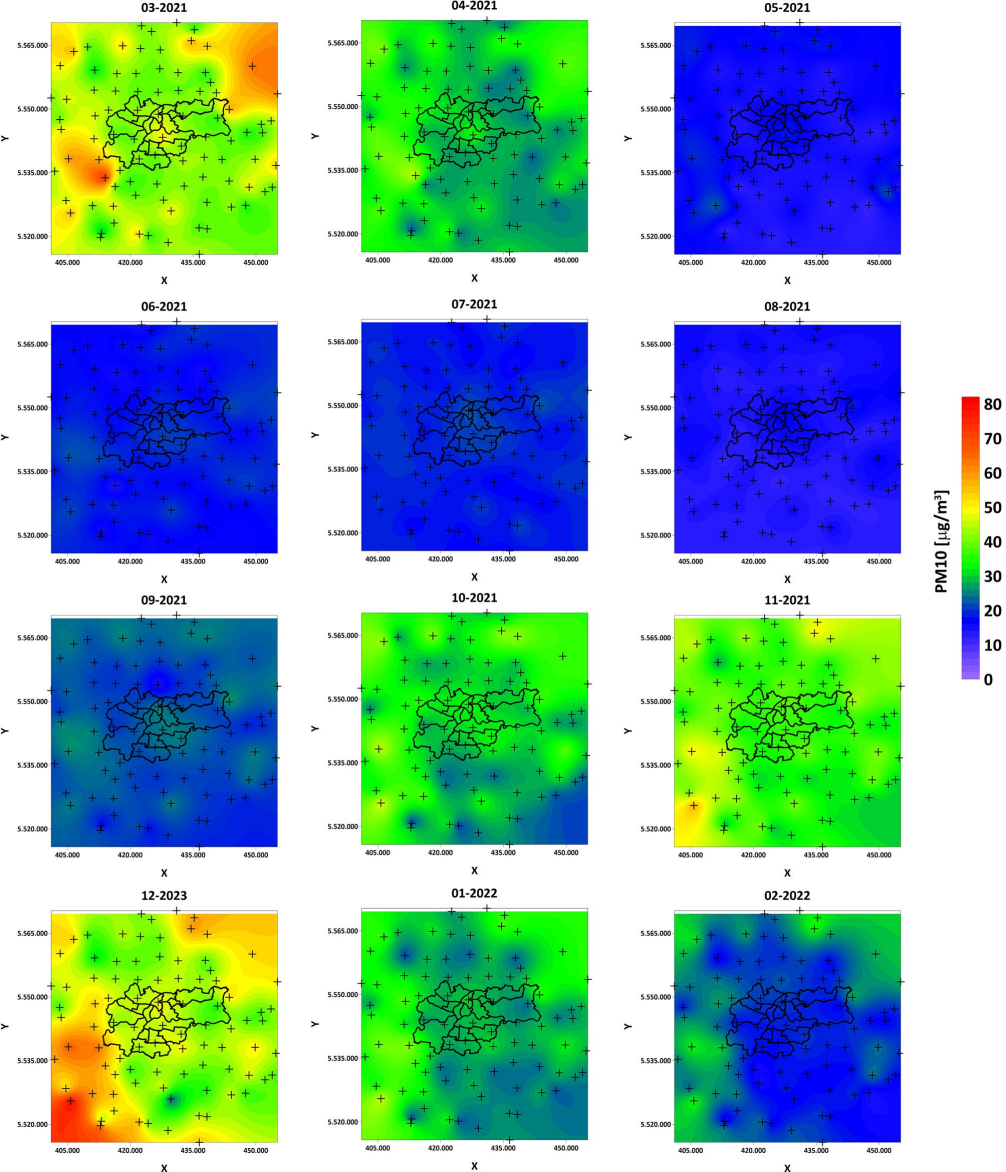
PM10 1-month average concentration maps for year cycle pattern analysis.

**Figure 14:**
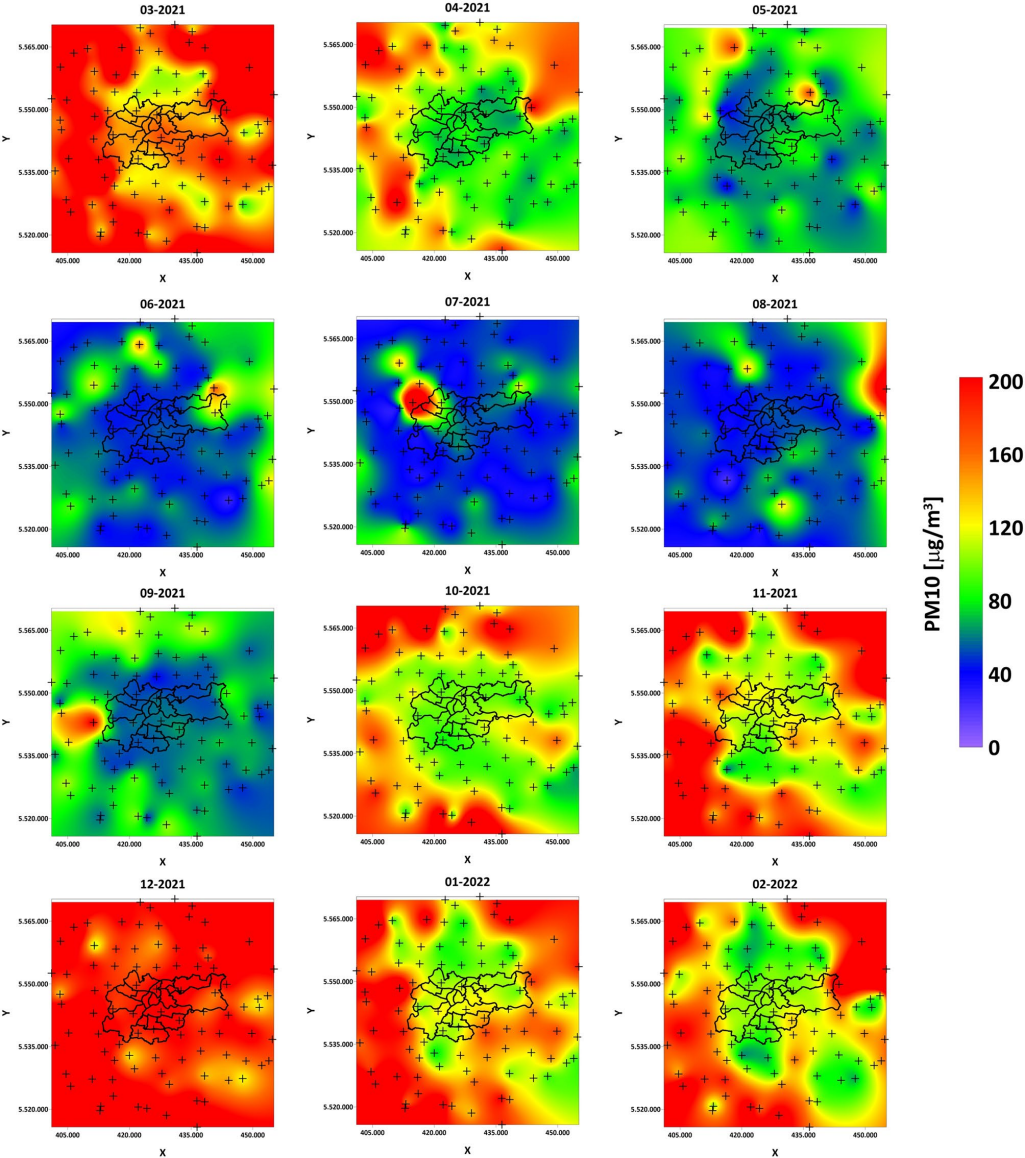
PM10 1-month maximum concentration maps for year cycle pattern analysis.

Figure 13 presents maps of the distribution of monthly average concentrations from March 2021 to February 2022. It is clearly visible that the months with very good air quality were May, June, July, August, and September. Interestingly, in the city of Krakow and the southeastern region, February 2022 also had very good air quality. In the remaining months, the air was of poorer quality, with the worst quality for March and December 2021. The most exposed to high average concentrations of the particular matter were the northeastern and southwestern regions. In the city of Krakow, pollution was distributed along the Vistula river, accumulating in the center of the old town. Generally, it can be stated that the pollution is distributed in this region along the southwest and northeast with a latitudinal distribution in the Krakow region. In the case of maximum monthly observations (Fig. 14), thanks to big data analysis, it is possible to observe a very important pattern of the maximum concentration distribution. A clear morphological barrier of the mountain range is visible, separating Krakow from the north and south. Previous observations, as well as our research, have led to the conclusion that Krakow’s location in a valley favors the accumulation of pollution, which is still true, but one should look at this problem differently by analyzing the distributions on the maps in Fig. 14. It can be seen that these barriers in some months allow isolating pollution outside Krakow (March, October, November 2021, January, and February 2022). The pollution is pushed into the city by the Vistula river valley. Unfortunately, there are months such as December, where in practically the entire studied region, the maximum concentrations are at the same, extremely high level. This pattern analysis allows for drawing an extremely important conclusion and confirming the accepted local nomenclature of the existence of the “obwarzanek krakowski” (a type of beagle meaning here circle around the Krakow). Morphological barriers on the one hand cause difficulties in the outflow of pollution, but on the other hand block the influx of pollutants from the surrounding regions located farther away.

### Air quality index

**Figure 15:**
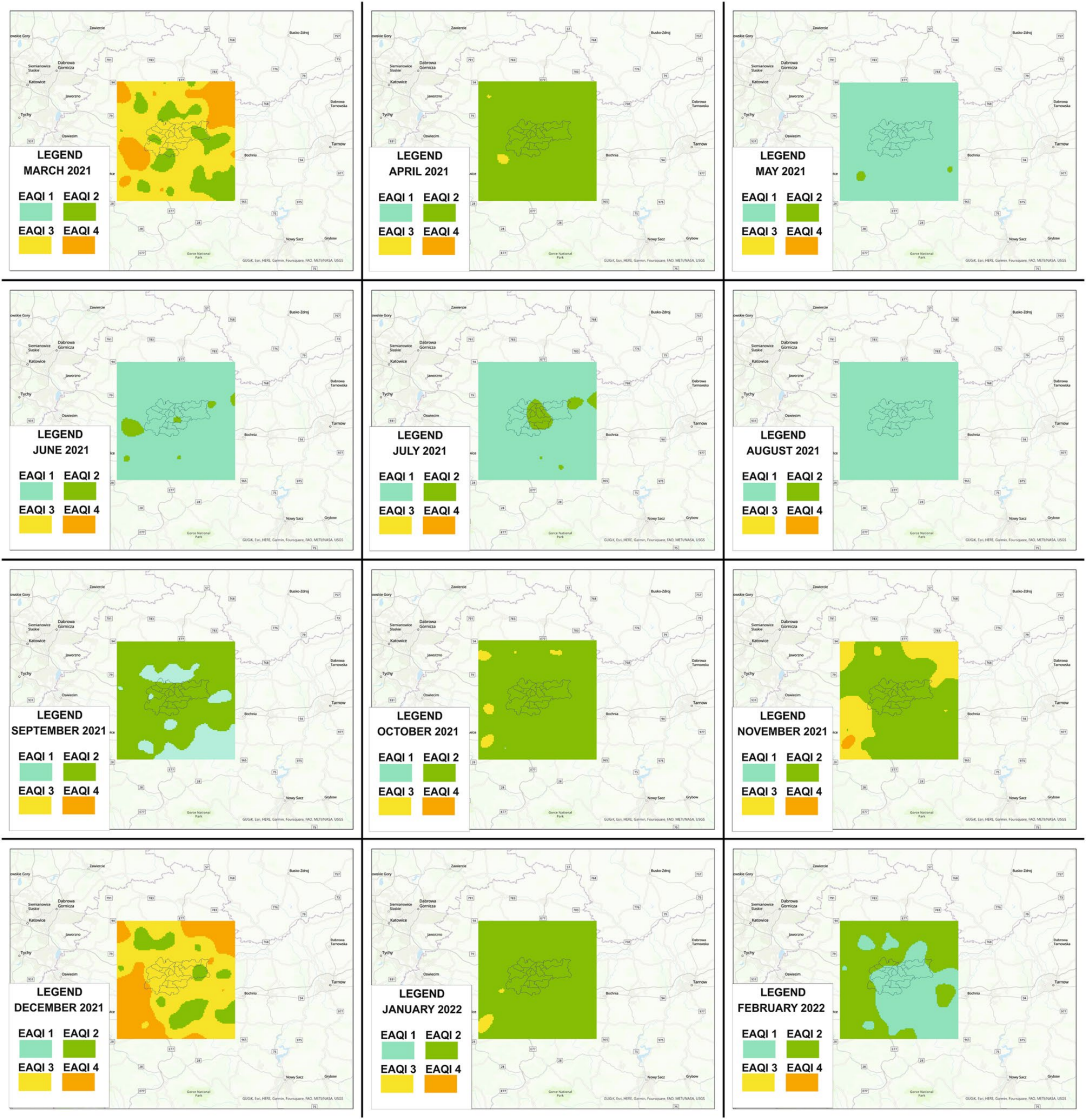
Air quality index clusters map in Krakow during the 1-year period.

The urban air pollution situation can be represented by different air quality indices. The European Air Quality Index (EAQI) is a measure of air quality across Europe’s regions provided by the European Environment Agency and the European Commission. EAQI is based on pollutant concentrations: PM10, PM2.5, O3, NO2, and SO2. We used ranges of PM10 values using the scale proposed by EAQI to determine the air quality at each point in the analyzed area. The bands of concentrations of PM10 and index levels are presented in Table 1. Figure15 shows air quality indices based on average PM10 concentrations in each month from March 2021 to February 2022.

**Table 1:** Bands of concentrations and index levels.

	Good	Fair	Moderate	Poor	Very poor	Extremely poor
Index value	1	2	3	4	5	6
PM10 [$\upmu$g/m^3^]	0–20	20–40	40–50	50–100	100–150	150–1200

As can be seen, air quality in the study area varies throughout the year. In general, two seasons can be clearly seen: the warm and the cold. It was possible to distinguish months in which the air quality, based on average concentrations in most or all of the analyzed area, was good (index value 1): May, June, July, and August. These are the months with the highest average daily air temperature. From October to April, air quality is worse (indices 2–4), with the exception of February, when there was good air quality in most of the analyzed areas. It was probably a situation associated with a more rapid increase in temperature and lower pressure. Analyzing spatially the air quality indices in the cold months in and around the area of Krakow, it can be concluded that in the municipalities around Krakow, the air is more polluted than within the city. The cleaner air in the city itself during the heating season is probably related to the local Air Quality Plan.

## Conclusions

The conducted research allowed for the examination of the annual relationship between meteorological factors and air pollution indicators in an urban area within a moderate climatic zone. By utilizing big data collection, processing, and analysis techniques, patterns and relationships of variables were able to be traced (in both time and space). The results of the study indicate a correlation between meteorological factors and air pollution in the urban area, particularly in relation to temperature. It was determined that the year is divided into two periods based on air pollution concentrations—a warm period and a cold one. In winter, fall, and spring, the strongest correlation was shown between temperature and PMx concentrations. With decreasing temperatures, an increase in emissions was observed. A decrease in temperature below 10 ^∘^C in this period caused an increase in emissions beyond acceptable concentrations. Two annual cycles were identified for temperature and dust, while three 4-month cycles were observed for humidity and four cycles of varying length for pressure. The analysis in terms of regions showed that the high-polluted areas are relatively stable in the cold months, along the southwest, and northeast axis, with a roughly parallel course along the Vistula valley. The analysis of patterns on surface distributions in different months showed that the distribution of maximum concentrations is primarily related to morphological barriers (elevated terrain). These barriers, on one hand, cause difficulties in leaving the pollutants from the city, but on the other hand, in some months, to some extent, protects the city from an even greater influx of pollutants from regions further away. An important observation is also the fact that through the analysis it was possible to detect high-emission episodes in the summer months, especially in the northeastern region, which may be related to the illegal burning of grass and agricultural land. The southwest region also showed a high level of emissions in the summer months, which may be related to industrial activities and transport. Overall, the results of the study indicate a clear relationship between meteorological factors (especially temperature) and air pollution in the Krakow area and reveal the need for further research and implementation of appropriate measures to reduce air pollution. These findings provide valuable insight into the dynamics of air pollution in the studied area and highlight the need for further research and implementation of appropriate measures to reduce air pollution. Our results, together with other activities such as the analysis of very local multi-pollution hot-spots^43^ can help local authorities with better and more sustainable planning. It is crucial to continue to investigate the complex interactions between meteorological factors, anthropogenic activities, and air pollution to develop effective strategies to improve air quality in urban areas.

## Data Availability

Publicly available datasets from Airly sensors were analyzed in this study and can be found here: (https://map.airly.org/, accessed on 19 Jan 2023). API documentation from Airly is available here: (https://developer.airly.org/en/docs, accessed on 19 Jan 2023). Publicly available datasets from E-OBS gridded datasets were analyzed in this study. This data can be found here: (https://www.ecad.eu/download/ensembles/download.php, accessed on 19 Jan 2023).
